# Functional roles of taurine, L-theanine, L-citrulline, and betaine during heat stress in poultry

**DOI:** 10.1186/s40104-022-00675-6

**Published:** 2022-03-10

**Authors:** Victoria Anthony Uyanga, Emmanuel O. Oke, Felix Kwame Amevor, Jingpeng Zhao, Xiaojuan Wang, Hongchao Jiao, Okanlawon M. Onagbesan, Hai Lin

**Affiliations:** 1 Department of Animal Science, College of Animal Science and Veterinary Medicine, Shandong Provincial Key Laboratory of Animal Biotechnology and Disease Control, Shandong Agricultural University, No. 61 Daizong Street, Tai’an, 271018 Shandong Province China; 2 Department of Animal Physiology, Federal University of Agriculture, P.M.B, Abeokuta, Ogun State 2240 Nigeria; 3 Farm Animal Genetic Resources Exploration and Innovation Key Laboratory of Sichuan Province, Sichuan Agricultural University, Chengdu, Sichuan China

**Keywords:** Amino acids, Antioxidant, Heat Stress, Immunity, Inflammation, Nutrition, Performance, Poultry

## Abstract

Heat stress (HS) is an important environmental stress factor affecting poultry production on a global scale. With the rise in ambient temperature and increasing effects of global warming, it becomes pertinent to understand the effects of HS on poultry production and the strategies that can be adopted to mitigate its detrimental impacts on the performance, health, welfare, immunity, and survival of birds. Amino acids (AAs) have been increasingly adopted as nutritional modifiers in animals to ameliorate the adverse effects of HS. They are essential for protein synthesis, growth, maintenance, reproduction, immunity, stress response, and whole-body homeostasis. However, HS tends to adversely affect the availability, transport, absorption, and utilization of these AAs. Studies have investigated the provision of these AAs to poultry during HS conditions, and variable findings have been reported. Taurine, L-theanine, and L-citrulline are non-essential amino acids that are increasingly gaining attention as nutritional supplements in HS animals. Similarly, betaine is an amino acid derivative that possesses favorable biological properties which contributes to its role as a functional additive during HS. Of particular note, taurine is negligible in plants, while betaine, L-theanine, and L-citrulline can be found in selected plants. These nutrients are barely found in feed ingredients, but their supply has been shown to elicit important physiological roles including anti-stress effects, anti-oxidative, anti-inflammatory, gut promoting, and immunomodulatory functions. The present review provides information on the use of these nutritionally and physiologically beneficial nutrients as functional additives to poultry diets during HS conditions. Presently, although several studies have reported on the positive effects of these additives in human and murine studies, however, there is limited information regarding their utilization during heat stress in poultry nutrition. Therefore, this review aims to expound on the functional properties of these nutrients, their potentials for HS alleviation, and to stimulate further researches on their biological roles in poultry nutrition.

## Background

In poultry production, several factors including the environment, nutrition, management, pathogens, and disease conditions can induce stress [1]. Since the 1800s, it has been reported that ambient temperature has risen by 1.0 °C, with the tendency to increase by 1.5 °C between 2030 and 2052 [2]. The increase in diurnal temperature sponsored by climate change and its antecedent of global warming on the earth’s surface has gained increasing concern and intensified investigations in the search for heat stress (HS) mitigation strategies. Alongside this, HS management has become a persistent challenge due to the rise in the human population, the higher number of production animals, and their higher metabolic activity due to genetic improvements [3].

In the tropical and sub-tropical regions of the world, HS is a major environmental stressor affecting poultry production. When there is a negative balance between the amount of heat generated by the animal and the amount of heat dissipated to its environment, these results to HS [4]. This negative balance is aggravated by other contributing factors such as sunshine, thermal irradiation, air temperature, relative humidity, housing condition, ventilation, stocking density, management conditions, environmental control systems, production systems, and animal characteristics (that is, the age, species, gender, metabolic rate, activity, and thermoregulatory mechanisms), which affects the animal’s responsiveness to HS [5–7]. In addition, HS regulates the concentration of free amino acids, altering the amino acid metabolism in poultry [8]. Consequently, during HS, there is an imminent need to devise useful strategies that would aid overcome stress effects in farm animals. Alongside environmental improvements to poultry housing, dietary manipulation is an important machinery that can contribute to alleviating the negative impacts of HS [9]. It had been revealed that despite improvements in management technology, nutritional manipulation is considered an effective strategy for HS alleviation in poultry [6]. Nutritional manipulation involves the inclusion/supplementation of functional additives (supplements) with beneficial properties to poultry diets [10]. It is an acceptable practice that involves the inclusion of vitamins, minerals, amino acids, phytogenes, growth promoters, antioxidants, nutraceuticals, herbs, probiotics, etcetera in poultry nutrition [11, 12].

Amino acids (AAs) serve as building blocks for the synthesis of proteins, bioactive peptides, and low-molecular weight metabolites, which regulate physiological functions in animals [13]. These AAs are useful candidates for feed adjustments since they play essential roles in animal metabolism and stress alleviation. The supplementation of functional AAs to low protein diets has been applied to promote livestock production and produce high-quality animal protein supply [13]. Asides from the conventional essential and nonessential amino acids, some non-standard amino acids (NSAA) have increasingly been utilized as feed additives in poultry nutrition. These NSAAs are typically non-proteinogenic, non-essential, and probable derivatives of secondary metabolites of the conventional AAs, with multi-beneficial effects. Although there are reports on AA utilization in poultry under stress conditions, there is limited information on the potentials of these functional NSAAs in poultry nutrition. This knowledge is considered important in the development of effective feeding strategies for poultry during HS conditions.

The NSAAs are involved in regulating the normal physiology of animals including affording protection from stressful conditions. Firstly is taurine, a non-protein AA whose requirement is likely increased during stress conditions to become a semi-essential AA [14]. Taurine is involved in numerous biological processes including anti-inflammatory, anti-oxidation, bile acid conjugation, membrane stability, osmoregulation, regulation of cellular calcium flux, and immunomodulation [15, 16]. Taurine has been shown to afford protective effects under stress models such as HS, endotoxin challenge, high stocking density, and in cases of toxicity [14]. Secondly, L-theanine, a naturally occurring AA in green tea leaves is widely used as a therapeutic agent, having functional roles in nervous regulation, antioxidation, and immunity [17]. Interestingly, studies have shown that L-theanine is a potent anti-stressor since it can decrease the concentration of stress hormones such as corticosterone, dopamine, and noradrenaline [18]. Thirdly, L-citrulline, a non-essential alpha-amino acid, is a crucial metabolite of the urea cycle [19] and is considered conditionally essential for gut functions [20]. It is involved in various physiological events, including arginine synthesis, nitrogen balance, anabolic processes, protein synthesis, growth and development, intestinal homeostasis, and muscle performance [21, 22]. Recent findings have expounded on the role of L-citrulline in affording thermotolerance to HS birds [23, 24]. Lastly is betaine which has been widely used as a highly valuable feed additive, and it is involved in various metabolic roles including protein synthesis, energy metabolism, anti-oxidation, osmoprotection, and methionine sparing [25]. Although not an amino acid, betaine is endogenously synthesized through choline metabolism, and as such, acts functionally as a vitamin or pro-vitamin [26, 27]. During HS, betaine supplementation provides beneficial effects against osmotic stress and dehydration, promoting survival [28]. Therefore, in the present review, these four feed additives are discussed explicitly. This article describes the functional roles and useful applications of these nutrients as anti-stressors and their biological actions in restoring health. Specifically, the beneficial application of these nutrients as feed supplements in poultry production and their role in alleviating the adverse effects of HS is discussed.

### Heat stress in poultry production

Poultry are endothermic homeotherms, since their body temperature is maintained within a narrow range of 40.0 to 42.2 °C [29]. High environmental temperature above the comfort zone initiates an irreversible cascade of thermoregulatory events that negatively affect their production and survival [30, 31]. Poultry birds are highly sensitive to high ambient temperatures since a large proportion of their body surface is covered with feathers and they do not possess sweat glands like most mammals for heat dissipation [32]. The thermoneutral temperature for most poultry species is around 18-20 °C, and at temperatures exceeding 30 °C, the birds are susceptible to HS [29]. The optimum temperature for laying hens lies between 19-22 °C and 18-22 °C for growing broilers [33]. In a bid to meet protein demand, increase feed efficiency, productivity, and disease resistance, poultry birds have been subjected to years of rigorous breeding and selection, which has yielded highly productive breeds for both the meat and egg production industries. Modern-day broilers have been intensively bred for high feed conversion efficiency, accelerated muscle growth, and high rate of mitochondrial metabolism but they possess a limited capacity for heat tolerance [34]. Laying hens produce high metabolic heat with their increased rate of egg formation, increasing their vulnerability to HS [35]. As such, present-day chickens are highly susceptible to HS since they have greater metabolic activity, high core body temperature, narrow comfort zones, and minimal heat dissipation [36].

HS is typically encountered as “acute HS”, which refers to the rapid exposure to high environmental temperatures and humidity over a short duration, or “chronic HS” which is regarded as exposure to high environmental temperature and humidity over a long duration, usually ranging from days to weeks [37, 38]. When birds are exposed to HS, they initiate the stress response which involves the immediate activation of the sympathetic nervous system (SAM) responsible for the “fight-or-flight response”, as well as the activation of the hypothalamo–pituitary–adrenal (HPA) axis, which is responsible for the secretion of glucocorticoids (mainly corticosterone in poultry) [39, 40]. Thus, the integration of the SAM and the HPA axis forms the two major components involved in stress response [41].

**Fig. 1:**
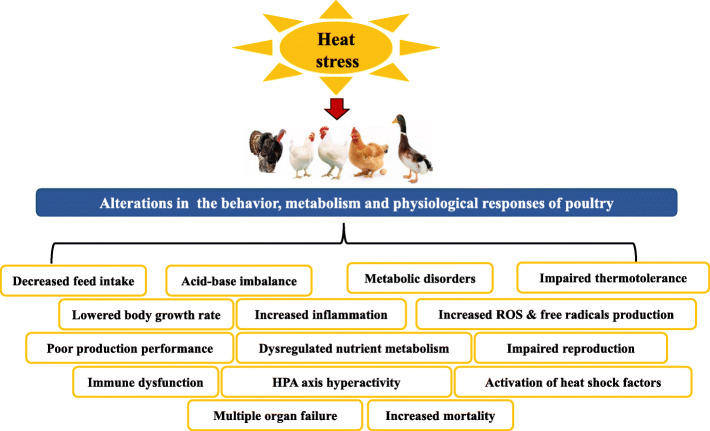
Effects of heat stress on the behavior and physiological responses of poultry

HS exposure causes severe changes to the physiology, nutrition, immunity, behavior, growth, and production traits of poultry, culminating in huge economic losses [31]. Under normal conditions, cellular ROS production occurs during ATP generation, however, HS exposure tends to induce the production of excessive free radicals, and a decrease in the enzyme activity and concentration of anti-oxidative parameters [42]. During chronic HS, increased ROS in skeletal muscle was evident due to increased oxygen consumption and the elevation of mitochondrial membrane potential at state 4 [43]. This imbalance causes oxidative stress, with resultant effects of lipid peroxidation and cellular damage [44]. More so, HS decreases the relative weights of lymphoid organs and alters immunoglobulin levels, which further predisposes the animals to infections [45]. Alongside having a diminished humoral immunity, immune cells such as lymphocytes, macrophages, and antibody responses are compromised in HS birds [32]. HS diminishes the growth performance and intestinal barrier function but promotes inflammation in birds [46]. Alhenaky [47] reported that HS increased serum corticosterone, systemic cytokines, lipopolysaccharide contents, and intestinal permeability to *Salmonella* spp. and endotoxins, causing increased mortality of birds. Elevated levels of corticosterone during HS alters the heterophil:lymphocyte ratio, immune functions, digestibility, glucose homeostasis, and nutrient metabolism [45, 48, 49]. Altogether, HS increases the body temperature, reduces feed intake and production indices, depresses immunity, acid-base imbalance, endocrine dysfunction, impairment in reproduction, blood pH and electrolyte imbalance, impairs nutrient digestibility, alterations to the gut microbiota, gastrointestinal dysfunctions, and increases mortality (Fig. 1, [24, 50, 51]). Detailed reviews on HS in poultry has been previously published [36, 38, 50, 51].

### Amino acids as nutritional modifiers during heat stress

Exposure to HS directly causes alterations (either an increase or decrease) in the levels of certain amino acids within the plasma, brain, and ileum tissues of poultry [52]. In a meta-analysis study, it was identified that HS increased the alanine, lysine, methionine, threonine, and serine but significantly decreased the cysteine, proline, and histidine concentrations in the plasma. Likewise, the brain concentration of glutamic acid, leucine, lysine, methionine, proline, threonine, valine, isoleucine, and histidine was increased by HS [52]. It is understood that during HS, birds increase their plasma volume to allow for heat dissipation via evaporative cooling. This may result in the dilution of blood components including AAs, lowering their concentration [53]. In addition, AA consumption and retention are lowered, and the dynamics of AA transporters are altered by HS in various tissues [54]. HS affects protein and amino acid metabolism via increased muscle protein breakdown during liver gluconeogenesis [55].

Excessive protein intake and metabolism exacerbates ionic imbalance and HS in poultry, thus it is recommended to offer low protein diets enriched with supplemented AAs to HS birds [56]. A strategy to promote poultry performance during HS is via improving their access to limiting nutrients while diminishing heat increment through the feed. It had been suggested that the supplementation of free AA should be in such a manner as to achieve an ideal balance beyond the requirement, which would promote effective protein supply and compensate for decreased feed intake during HS [57]. In addition, Suganya et al. [58] reported that the proportion of critical amino acids supplied during HS should be increased by 5-10% compared to normal conditions. It was shown that feeding low protein diet (207, 193.5, and 175.5 g/kg at the starter, grower, and finisher phases (90% of the Ross 308 recommendations for CP)) fortified with AAs including valine, lysine, methionine, and threonine, supported the growth of cyclic HS broilers equivalent to birds fed with a standard CP diet [44]. Altogether, studies have shown that supplying adequate amounts of both essential and non-essential AAs can alleviate the detrimental effects of HS and promote production performance in poultry [59, 60].

### The utilization of non-standard amino acids and betaine as functional additives during heat stress

#### Taurine

**Fig. 2:**
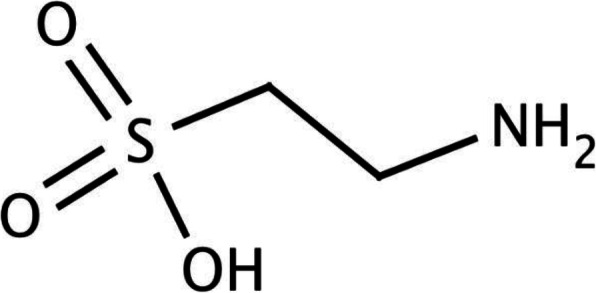
Chemical structure of Taurine

Taurine (2-aminoethanesulfonic acid) is a sulfur-containing β-amino acid [Fig. 2], which is highly abundant in the brain, heart, liver, kidney, and skeletal muscles of animals [61, 62]. It has been implicated in a myriad of functions such as cellular homeostasis, stress response, anti-oxidation, neuroprotection, energy metabolism, ion movement, calcium signaling, osmoregulation, cytoprotection, and mitochondrial functions [16, 61, 63, 64]. The utilization of taurine to improve poultry growth and performance has proved quite variable and inconclusive [65–67]. Interestingly, taurine is widely gaining research interest following the discovery that taurine inclusion in poultry nutrition elicits protective effects under different stressors including environmental, nutritional, technological, and biological stressors [1, 14].

The utilization of taurine during HS conditions in poultry has yielded significant and remarkable findings with regards to HS adaptation, thermotolerance, anti-inflammation, antioxidation, and improved production performance (Table 1). It was shown that pre-feeding broilers with taurine (0.1% taurine) from 2 weeks of age, improved the final BW during HS (34 °C, 60% RH for 3-5 weeks) [68]. In contrast, some studies have reported that taurine supplementation did not alleviate HS depression of the BW, ADFI, and ADG in broilers [70, 72]. In 6 d old chicks, central administration of taurine induced anorexia and dose-dependent hypothermia via a GABAAR-dependent mechanism under thermoneutral conditions, whereas during HS, central taurine increased the glucose and uric acid levels, but decreased the sodium and calcium concentrations [69]. A study on quails fed with dietary taurine (2.5 or 5 g/kg of diet) reared under HS (34 ± 2 °C, HS for 8 h/d; 12 weeks) showed that higher taurine concentrations improved the feed intake, egg production, and apparent digestibility [74]. Also, taurine decreased the MDA concentrations but upregulated the gene expressions of nutrient transporters in the ileum such as *PEPT1, EAAT3, CAT2, SGLT1, SGLT5, GLUT2,* and *GLUT5* during HS conditions [74]. Taurine alleviated HS impairment of the intestinal morphology in broilers by increasing the villus height but decreased the crypt depth in broilers' intestines [70]. Moreover, HS increment of intestinal hormones such as SS, PYY, ghrelin, and CCK in the duodenum, was further amplified with taurine supplementation, resulting in lowered feed intake due to the excessive release of these anorexic gut hormones [70]. These intestinal anorexic hormones inhibit appetite via impeding intestinal movements and inducing satiety [98]

**Table 1:** Summary of the functional roles of taurine, L-theanine, L-citrulline and betaine in poultry species

Animal model	Dosage and administration	Stress model/study design	Major findings	Reference
**Taurine**				
One-day-old Ross broilers	0.1% taurine was added to drinking water from 2 weeks of age	The treatments consisted of control birds which were reared at 24 °C and HS group at 34 °C, 60% RH from 3 to 5 weeks of age	Taurine lowered the gene and protein expressions of heat shock proteins in the liver and muscle tissues of HS broilers	[68]
Julia layer chicks (6 days old)	Taurine was dissolved in 0.85% saline containing 0.1% Evans blue for intracerebroventricular (ICV) injection	Chicks were ICV injected with either saline or 5 μmol taurine and exposed to either 35 ± 1 °C or 30 ± 1 °C for 60 mins in temperature controlled chambers	Central taurine induced dose-dependent hypothermia and inhibited food intake under control temperature. During heat stress, central taurine altered the plasma metabolites suggesting that brain taurine may supply energy and protect against oxidative stress during high temperature in chicks.	[69]
Male Arbor Acres broilers	Basal diet was supplemented with 5 g/kg taurine	Broilers were raised at thermoneutrality, 22 °C or under consistent HS at 32 °C, 55 ± 5% RH from 28 days of age	Taurine had no positive effects on the growth performance of HS birds but it alleviated the adverse impacts of HS on the jejunal morphology, increased duodenal somatostatin and peptide YY hormones, and upregulated the intestinal expression of appetite related genes in HS broilers	[70]
Male Arbor Acres broiler chicks	Broilers were fed the basal diet supplemented with 5.00 g/kg taurine	At 28 days old, broilers were subjected to thermoneutral (22 °C), consistent HS, (32 °C), or HS + Taurine (consistent 32 °C, basal diet + 5.00 g/kg taurine) and 55 ± 5% RH for a 14-day trial	Taurine decreased reactive oxygen species and malonaldehyde but increased nuclear factor erythroid 2-related factor 2 (Nrf2), NAD(P) H quinone dehydrogenase 1 and heme oxygenase 1 expression in HS broilers. Taurine also alleviated mitochondrial damage and improved the breast meat quality in chronic HS broilers	[71]
Male Arbor Acres broilers	Broilers were fed basal diet supplemented with 5.00 g/kg taurine from 28 days old	Broilers were randomly distributed to positive control (22 °C, basal diet), HS, (constant 32 °C, basal diet), or heat stress + taurine, 55 ± 5% RH for 14 d.	Taurine supplementation did not alleviate the high cloacal temperature in chronic HS broilers. Taurine improved the carcass by facilitating lipolysis for energy, enhancing protein synthesis, and suppressing protein degradation in the breast muscles in HS broilers	[72]
Male Arbor Acres broilers	Experimental diet was supplemented with 5 g/kg taurine	Broilers were grouped as control group (22 °C), HS group (32 °C) and HS + taurine fed group	Taurine supplementation attenuated breast muscle loss induced by chronic HS via reversing endoplasmic reticulum stress induced apoptosis and suppressing protein catabolism. Taurine moderated the decreases in breast muscle mass and yield in chronic HS broilers.	[73]
Laying Japanese quails (*Coturnix coturnix japonica)* at 5 weeks of age	Taurine was supplemented at 2.5 or 5 g/kg diet	Animals were housed in temperature-controlled rooms for 12 weeks at either 22 ± 2 °C for 24 h per day considered as thermoneutral or under 34 ± 2 °C for 8 h per day, followed by 22 °C for 16 h considered as heat-stress (HS).	Taurine supplementation, especially at higher dose(5 g/kg) improved the production performance, nutrient digestion, and ileal nutrient transport in HS quails	[74]
**L-Theanine**				
Male WENS yellow-feathered broilers	Birds received a corn soybean meal basal diet in mash form or a basal diet supplemented with 800 mg/kg L-theanine	At 24, 25 and 26 days of age, birds were intra-abdominally injected with 0.2 mL sterile saline or LPS (*Escherischia coli* serotype O127:B8) dissolved in saline at adequate doses of 600 mg/kg BW	L-theanine exerted a protective role on the growth performance of LPS-challenged broilers and attenuated LPS-induced immune stress	[17]
Arbor Acre broilers	Basal diets were supplemented with different concentrations of L-theanine at 0, 100, 200 and 300 mg/kg feed	Experimental birds were divided into 4 treatments groups of control and 3 levels of L-theanine supplementation	Supplementation of L-theanine up to 200 mg/kg enhanced the growth performance, meat quality, immune response, and anti-oxidant status of broilers but reduced the total serum cholesterol levels. However, higher dose up to 300 mg/kg L-theanine may pose deleterious effects on the performance and health of birds.	[75]
Arbor Acre broilers	L-theanine was mixed in basal diets at different concentrations at 0, 100, 200, and 300 mg/kg feed	Experimental treatments included control (basal diet); basal diet + 100mg L-theanine/kg diet; basal diet + 200mg L-theanine/kg diet; and basal diet + 300mg L-theanine/kg diet	L-theanine promoted immune and growth responses by favoring the abundance of beneficial gut microbes and downregulating the expression of inflammatory mediators	[76]
Yellow-feathered broilers	L-theanine was provided at four different levels of 100, 200, 400 and 800 mg/kg	Broilers were grouped as the control group (basal diet); antibiotic group; and four L-theanine test groups at different levels of 100, 200, 400 and 800 mg/kg diet. The test period was 49 d.	L-theanine had no adverse effects on the production performance and immune organ index of yellow feather broilers at different production stages	[77]
Egg laying chickens	L-theanine was provided as 200 mg/kg in the basal diets	The ambient room temperature of experimental chickens was controlled at 32 ± 3 °C, and 70% RH. The trial period lasted for 28 d	L-theanine had no significant effect on the growth performance of chickens. However, L-theanine increased the catalase activity but reduced malondialdehyde content in various tissues providing antioxidant effects	[78]
Chaohu ducks	Basal diets were supplemented with 0 (control), 300, 600, 900 and 1500 mg/kg of L-theanine	Room temperature was between 27 to 36 °C, with 70% RH. Lighting period was 23 h/d and the trial lasted for 28 d	L-theanine yielded significant improvements in immune function and jejunum morphology and antioxidant capacity of ducks. The optimum inclusion levels of L-theanine was 600 to 900 mg/kg based on the current experimental condition	[79]
Male Ross 308 broilers	Dietary L-theanine was supplemented at 600 mg/kg of diet	Broilers were subjected to 3 protocols of 0-h transport (control group), 3-h transport, and 3-h transport + dietary L-theanine supplementation	L-theanine alleviated transport-stress-impairment of immune organ indexes and meat quality of broilers. L-theanine reversed the detrimental effects of transport stress on muscle antioxidant capacity and glycolysis metabolism.	[80]
**Betaine**				
Hens and roosters of Mandarah strain	Dietary supplementation of 1000 mg/kg betaine	Control conditions: 22–24 °C; 45–55% RH or Chronic HS at 38 ± 1 °C; 55–65% RH from 11:00 to 15:00 h for 3 weeks	Betaine supplementation during chronic HS improved the body weight gain, survival rate, laying rate, egg mass and feed intake	[81]
Mandarah (a dual-purpose breed) chickens	Basal diet supplemented with 1000 mg/kg of betaine	Thermoneutral conditions of 22–24 °C, 45–55% RH or chronic HS (38 ± 1 °C; 55–65% RH) for three successive days a week, from 11:00 to 15:00 h	Betaine supplementation alleviated the adverse effects of chronic HS by improving the semen characteristics, fertility, physiological, haematological indices, antioxidant status, wellbeing, and intestinal DNA functions of breeder roosters	[82]
Male White Ross breed broiler chickens	Daily oral administration of betaine hydrochloride at 250 mg/kg for 42 d	Hot dry season having dry-bulb temperature (28.33-35.67 °C), relative humidity (69.0-93.0%), and THI (27.85-36.1)	Betaine and its co-administration with ascorbic acid decreased fearfulness in birds and increased antioxidant enzymes activities of SOD and GPx activity in broiler chickens	[83]
Female Japanese quails	Dietary supplementation of betaine at 2 g/kg of feed	Dry season conditions with highest mean dry-bulb temperature of 32.0 to 32.1 °C, highest mean THI of 85.4­-85.5 and highest mean RH of 79.6%	Dietary enrichment with betaine and ascorbic acid improved activities of serum sex and stress hormones, and erythrocytic parameters of Japanese quails during thermally stressful dry season.	[84]
Day-old broiler chicks	Betaine (Betafin.), was administered at the dose rate of 50 g/50 kg of feed from the first day of experiment	Hot summer season (average temperature, 34.6 °C) where HS birds were managed without using desert cooler	Betafin improved the growth performance and immunity of birds during heat stress	[85]
Ross 308 male broiler chickens	1 g/kg feed of betaine was added in powder form on top of the basal diets	HS chickens were housed in a chambers at 34 °C for 8 h (9:00–17:00 h).	Dietary betaine improved the growth performance (ADG, EPI, FCR) and humoral immunity against NDV and infectious bronchitis virus in heat-stressed broilers	[86]
Yellow-feathered male broilers (Huaixiang chickens)	Basal diets supplemented with 500, 1000, 2000 mg/kg betaine	Birds were exposed to thermoneutral conditions of 26 ± 1 °C or cyclic HS of 32 ± 1 °C for 8 h/d from 9:00 to 17:00 h with 65–75% RH	Dietary betaine alleviated the impacts of long term HS on the growth performance, digestive function, and carcass traits in indigenous yellow-feathered broilers.	[87]
Meat-type ducks	Betaine was supplemented at 700, 1000 and 1300 ppm betaine	From 22 to 42 days of age, heat wave was applied at 11:00 to 17:00 h, 33 to 43 °C, and 70% RH, followed by maintaining at 22 to 26 °C from 17:00 to 11:00 h, 50% RH	Betaine supplementation had beneficial effects on the short chain fatty acid levels, hematological parameters, and body weight of heat stressed ducks.	[88]
Hy-line Brown laying hens	3.0 or 6.0 g/kg of purified betaine was supplemented to the basal diet at the expense of celite.	Hens were raised during the hot season with 25.8 ± 2.0 °C average daily room temperature, 74.8 ± 7.3% RH, and heat stress index of 76	Dietary betaine improved the hen-day egg production, decreased the broken and shell-less egg production and selectively modified the jejunal tight junction-related genes in laying hens raised under hot environment	[89]
Male broiler chickens (Cobb × Cobb)	Betaine (Betafin®) was supplied in the drinking water (50 g/kg) or feed (100 g/kg)	Broilers were subjected to 34 ± 1 °C, 75% RH for 4 h in environmental chambers at 35 day, then increased to 36 ± 1 °C for 4 h/d from d 36 to 41.	Study revealed that birds supplemented with betaine via drinking water had better resistance against high ambient temperatures than birds fed betaine in diets	[28]
**L-Citrulline**				
Ross 308 broiler chickens	Dietary supplementation with 1% L-Citrulline of basal diet	Broilers were subjected to two environments, either thermoneutral at 24 °C or HS at 35 °C for 5 h, 60% RH	L-Citrulline affected the body temperature, antioxidant status, heat shock response and nitric oxide regeneration of broilers during HS and at thermoneutrality	[24]
Hy-Line Brown laying hens	Dietary addition at 0.25%, 0.5%, and 1% L-citrulline to basal diets	Summer season with average daily minimum and maximum temperatures of 25.02 °C and 31.01 °C	Dietary L-Citrulline did not influence the production performance, and rectal temperature of laying hen. L-Citrulline modulated systemic arginine metabolism, nitric oxide synthesis, and antioxidant defences of laying hens	[90]
Hy-Line Brown laying hens	Diets were offered as a reduced protein diet deficient in Arginine supplemented with 0.35% L-Citrulline at the expense of wheat	Birds were fed commercial diets 16 to 20 weeks of age and experimental diets started from 21 to 40 weeks of age.	Supplementation of either L-Citrulline to reduced protein diets did not affect the egg quality, protein and energy digestibilities of hens but tended to increase the Haugh unit and lower the shell breaking strength of eggs	[91]
KUB Chicks	Oral administration of L-citrulline at 3.75, 7.5 and 15 mmol/kg body weight.	5 days old chicks received L-Citrulline orally	L-Citrulline did not influence the feed intake, body temperature or plasma metabolites in chicks.	[92]
Male layer chicks (Julia)	Chicks received oral administration of L-Cit (15 mmol/10 mL/kg body weight) as single or double doses	Birds were exposed to HS (35 ± 1 °C) or thermoneutral temperature (30 ±1 °C) for 180 mins.	Single L-citrulline administration caused persistent hypothermia and lowered plasma glucose without affecting food intake. Dual administration of L-Citrulline afforded thermotolerance without a significant change in plasma nitric oxide of chicks	[23]
Male layer chicks (Julia)	L-Citrulline was administered as i.c.v. injection at 1 μmol/10 μL dosage. Orally administered L-citrulline was at 3.75, 7.5 or 15 mmol/10 mL/kg body weight	Exp. 1 was an intracerebroventricular (i.c.v.) injection while Exp. 2 was the oral administration of L-citrulline	Central citrulline did not alter body temperature, whereas, peripheral L-citrulline had a hypothermic effect in a dose responsive manner. Rectal temperature was decreased at 30, 60 and 120 mins after injection of the highest dose of L-Citrulline.	[93]
Male layer chicks (Julia)	Oral administration of watermelon rind extract (1.6 mL) or L-Cit (15 mmol/10 mL)	Chickens were treated with dual oral administration of (1.6 mL) watermelon rind extract or L-Cit (15 mmol/10 mL and exposed to high ambient temperature (35 ± 1 °C, 2 h) for 120 mins	Watermelon rind extract reduced rectal temperatures under control and heat stressed conditions in a similar fashion as high L-citrulline treatment	[94]
Male layer chicks (Julia)	Watermelon rind dried powder (WRP) was mixed with commercial starter diet to prepare a 9% WRP mash diet.	WRP mash diet was fed to 3- to 15-day-old chicks	Chronic supplementation of the WRP mash diet increased plasma L-citrulline levels, but did not affect the body temperature in chicks	[95]
Layer chicks	Oral administration of either a medium containing L-Citrulline producing live bacteria and 277 mmol/L L-Citrulline or an equimolar amount of L-Citrulline	In Exp. 1, chicks were orally administered treatments at 7-day-old and in Exp. 2, chicks were subjected to chronic treatment from 7 to 13 days of age	Acute or chronic administration of the media containing L-citrulline-producing live bacteria decreased the rectal and surface body temperatures of chicks, but an equimolar amount of L-citrulline elicited no changes	[96]
Ross 308 cockerels	L-citrulline was supplemented to low protein deficient in Arg at two levels of 0.238% and 0.476% L-Citrulline	Dietary treatments included eight groups assigned as normal-protein diet; low-protein diet deficient in Arginine (LP) and LP with two levels of either Arginine (0.238% and 0.476%), guanidinoacetic acid (0.309% and 0.618%) or Citrulline (0.238% and 0.476%).	L-Citrulline supplementation to low protein diets increased the body weight gain, carcass yield, bone length, diameter and ash but did not increase the ileal energy or nitrogen digestibility	[97]

Taurine supplementation at 5.00 g/kg of basal diet was investigated on the growth performance and carcass characteristics in chronic HS broilers. Taurine increased the breast muscle proportion but decreased *MAFbx, MuRF1, ACC,* and *M-CPT1* genes in breast muscle during HS. Also, taurine enhanced lipase activity in the abdominal fat, and serum NEFA concentration suggesting that under chronic HS, taurine supplementation can significantly promote lipolysis and protein synthesis, while suppressing protein degradation in breast muscles tissues [72]. Taurine supplementation to HS broilers (32 °C, basal diet + 5g/kg for 2 weeks) attenuated HS-induced breast muscle loss via suppressed protein catabolism but significantly improved the breast muscle mass and yield [73]. Additionally, taurine had been shown to influence lipid metabolism in broilers via increasing the hepatic expression of *AMPKα, SIRT1,* and *CPT-1* but decreased *SREBP-1* expression, and further altered the serum TG, TCHO, and hepatic lipase activity [99].

**Fig. 3:**
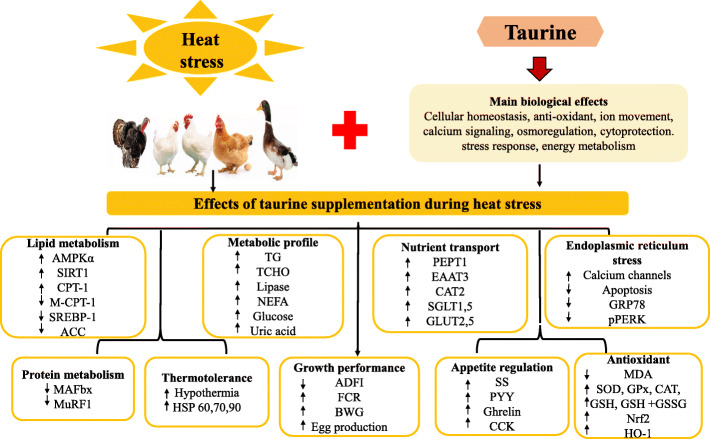
Effects of taurine supplementation during heat stress in animals

In several studies, taurine acts to attenuate inflammation and oxidative stress. At the molecular level, taurine modulated endoplasmic reticulum (ER) stress, Ca^2+^ homeostasis, and neuronal activity [62]. HS can induce oxidative damage and mitochondrial impairment in tissues, however, taurine is a potent antioxidant that functions to promote the antioxidant defense mechanisms and attenuate ROS generation [71]. Taurine is a non-enzymatic, free radical scavenger that possesses antioxidant properties arising from its sulfur moiety [100, 101]. It is known that apart from scavenging ROS directly, taurine can also act to enhance the cellular antioxidant enzyme activities for SOD, GPx, and catalase [102]. Taurine supplementation reduced myocardial MDA, preserved GSH levels, and normalized the GSH + GSSG levels, preventing damage from oxidative stress [101]. In addition, taurine activated the Nrf2 pathway (a major regulator of cellular defenses against oxidative stress) and enhanced *HMOX1* expression to combat redox imbalance and alleviate oxidative impairment [103]. Taurine supplemented broilers exposed to chronic HS (consistent 32 °C, 55 ± 5% RH; basal diet + 5.00 g/kg; 14 d) showed decreased ROS and MDA production. Taurine alleviated HS-induced structural damage of the mitochondria and upregulated *Nrf2, NAD(P)H,* and *HO-1* expression in the breast muscle, affording mitochondrial protection and improving the redox status of HS broilers [71]. Also, during HS, taurine reversed ER stress by mediating the expressions of Ca^2+^ channels, stress factors, and apoptotic factors in the breast muscle of broilers [73]. Additionally, taurine can afford thermotolerance by regulating the expression of heat shock genes and proteins [14]. Taurine downregulated HS-induced increment in the mRNA expression of *HSP 60, 70, *and* 90* and protein expressions of HSP 60 and HSP 70 in hepatic tissues of broilers [68]. Summarily, the protective effects of taurine during HS are depicted in Fig. 3.

#### L-Theanine

**Fig. 4:**
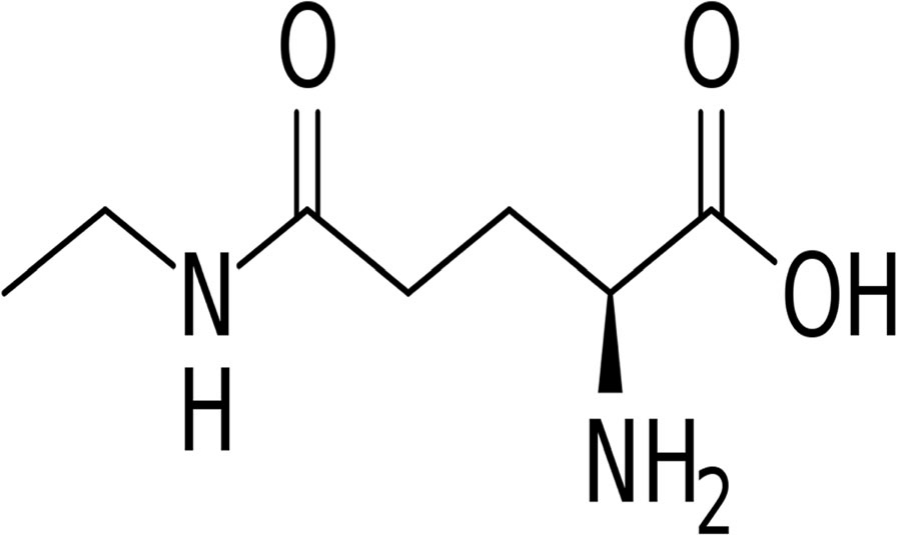
Chemical structure of L-theanine

L-Theanine, chemically known as 2-amino-4-(ethylcarbamoyl) butyric acid, is a non-protein amino acid (Fig. 4) that can be obtained from glutamic acid [104]. It is naturally found in tea plants and constitutes a major component of green teas (*Camellia sinensis*) [105]. It is synthesized in the roots and accumulates in tea leaves, with an average content of 1.2 to 6.2 mg/g fresh weight, 1 to 2.5% of total leaf weight, and represents about 50% of total free amino acids in teas [106]. Research on L-theanine has gained attention due to its numerous health benefits, including its growth promotion, anti-apoptosis, anti-oxidation, anti-stress, anti-anxiety, anti-carcinogenic, neuroprotection, antimicrobial, immunomodulation, and anti-inflammatory functions [18, 107–110]. It can cross the blood-brain barrier to afford neuroprotection against oxidative stress [111]. L-Theanine has been reported as basically non-toxic, with no adverse effect on the physiological and histopathological characteristics in mammals [105]. It is widely used as a functional ingredient and dietary supplement for humans or animals [112].

**Fig. 5:**
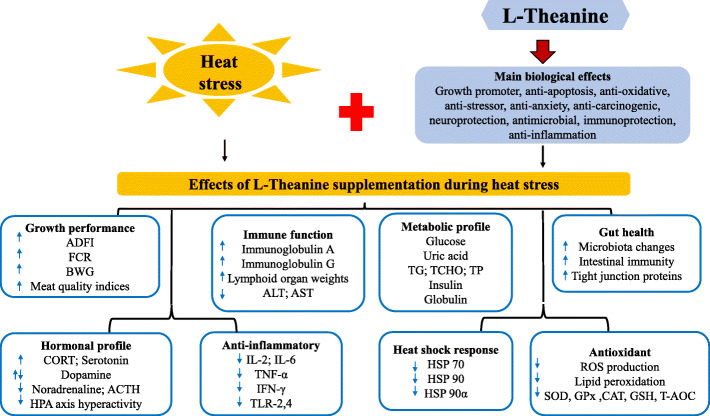
Effects of L-Theanine supplementation during heat stress in animals

In human and murine models, L-theanine has been applied using various acute and chronic stress conditions [113]. L-Theanine elicits anti-stress effects by attenuating the activation of the sympathetic nervous system [114]. Serum corticosterone level was lowered in L-theanine treated rats subjected to water immersion stress [115]. In mice subjected to chronic restraint stress, L-theanine treatment (2 and 4 mg/kg) elicited protective effects by reversing the extent of cognitive impairment, oxidative damage, and the abnormal concentration of corticosterone, norepinephrine, and dopamine in the serum and brain tissues [116]. In poultry, variations in circulating corticosterone serve as physiological stress indicators [18], since it increases during exposure to stress stimuli, however, prolonged elevation in plasma corticosterone and activation of the HPA axis results in deleterious consequences [117]. Therefore, these stress and fatigue-relieving effects of L-theanine pose potential benefits in poultry production (Fig. 5) [51].

Presently, there are limited reports on L-theanine application in livestock and poultry production [18], and a handful on its utilization for HS alleviation in poultry, creating an existing knowledge gap in poultry nutrition researches. Dietary supplemented L-theanine optimized at 600 to 900 mg/kg in duck’s feed had positive effects on the growth performance, antioxidant capacity, and jejunal morphology of birds [79]. Also, L-theanine improved the protein metabolism, immune function, and antioxidant capacity in the small intestine and liver of roosters [118]. Serum metabolites such as GLU, UA, TG, TCHO, low-density lipoprotein cholesterol, insulin, IL-2, and IL-6 were reduced, while the total protein, globulin, IgA, and IgG were increased [79]. Supplementation of L-theanine in broiler’s diet improved the expression of tight junction proteins including ZO-1, occludin, and claudin-3 [76]. Also, it was shown that L-theanine positively influenced gut health and growth responses in broilers via two routes; firstly by favoring an increase beneficial microbes (*Lactobacillus*), and reducing pathogenic microbes (*Clostridium*), and secondly by mediating the intestinal immune response via downregulating* TLR-2, TLR-4, TNF-α, IFN- γ,* and *IL-2* genes [76]. In addition, L-theanine with L-glutamine synergistically alleviated intestinal stress by increasing the intestinal villi length and crypt depth during *E. coli* infection [119].

Supplementing L-theanine to young roosters promoted HS tolerance (35 ^o^C, 70% RH), growth performance, and immune organ index at concentrations above 200 mg/kg [118]. In laying chickens exposed to HS, L-theanine showed no significant effects on the growth performance, but promoted the CAT activity and decreased the MDA content in various tissues and organs [78]. In another study, it was shown that dietary L-theanine (600 mg/kg) supplementation improved the FCR and BW of broilers, and mitigated transport stress by increasing the lymphoid organ index (thymus, spleen, and bursa of Fabricius), muscle antioxidant activity (T-AOC, CAT, and GSH-PX) and meat quality indices (decreased drip loss, muscle MDA, PC, and lactate contents) [80]. Studies from other animals also validate the efficacy of L-theanine as an anti-stress agent. L-theanine (100 or 200 mg/kg BW) treatment in mice subjected to whole-body HS (42 °C, 60% RH for 2 h) prevented the upregulation of heat shock proteins (HSP70, HSP90, and HSP90α), minimized heat-induced liver damage, oxidative stress (GSH, T-SOD, CAT, and MDA levels), inflammatory responses, and reversed HPA axis hyperactivity (lowered plasma ACTH and CORT levels) [120]. It is understood that L-theanine antagonism of the glutamate receptor allows for its inhibition of the HPA axis hyperactivity [120]. In HS mice, L-theanine increased the feed intake and body weight, enhanced SOD, CAT, GSH-Px activities in the liver and jejunum, and reduced the serum contents of ALT, AST, TNF-α, IL-6, and IFN-γ [121].

L-Theanine elicits its anti-oxidative effects via attenuating ROS production, decreasing lipid peroxidation, and promoting glutathione concentration [122]. It can regulate both the non-enzymatic and enzymatic anti-oxidative activities via transcriptional, post-transcriptional, and post-translational mechanisms [123]. In a rat model of cerebral ischemia/reperfusion injury, L-theanine treatment afforded neuroprotective effects by inhibiting *HO-1* expression but activated the ERK1/2 pathway in the hippocampus [124]. In another study conducted to investigate the protective effects of L-Theanine in D-galactose-induced senescent rats, it was revealed that L-theanine reversed the imbalance in oxidative stress (increased SOD, CAT, GPx, lowered MDA), inflammatory responses (lowered *IL-1β, IL-6, TNF-a*; but increased *IL-4 *and* IL-10*), liver aging and inhibited the phosphorylation of FoxO1 and NF-κB (p65) in liver tissues [125]. L-theanine supplementation downregulated the colonic mRNA expression of *iNOS, COX-2,* and *TLR-2/-4/6/-9*; upregulated *ZO-1* and claudin-1 expression; decreased pro-inflammatory factors (*IL-1β, IL-6, TNF-α, MCP-1, *and* MPO)* and also reversed the imbalance in GSH and MDA elicited during DSS induced colitis [126]. Altogether, it is evident that L-theanine improves intestinal absorption, reduces inflammation and oxidative damage, and attenuates tissue and organ damages during HS (Table 1).

#### L-Citrulline

**Fig. 6:**
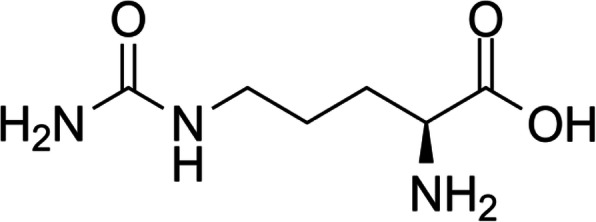
Chemical structure of L-citrulline

L-Citrulline (C_6_H_13_N_3_O_3_)^6^, a neutral, non-essential, non-protein amino acid, is a metabolic intermediate in the urea cycle (Fig. 6) [19, 127]. Recent evidence has pointed out its almost critical role in pharmaconutrition [128]. In several studies, L-citrulline has been demonstrated in protein synthesis, intestinal homeostasis, nitrogen balance, growth and development, anti-oxidation, gut barrier function, muscle performance, intestinal digestion and absorption, renal function, exercise performance, blood pressure, vasodilation, anti-inflammation, L-arginine synthesis, and nitric oxide production [22, 127–131]. L-Citrulline is naturally occurring in living organisms, but it is largely found in watermelon (*Citrullus vulgaris*), and variable quantities are present in cucumbers, pumpkins, squash, bitter melons, muskmelons, and gourds [129]. It can be derived directly from L-arginine through the action of nitric oxide synthases resulting in nitric oxide production or via conversion by arginase into ornithine in the urea cycle to deliver L-citrulline [21, 132]. L-Citrulline has been described as well tolerated, with no side effects [133].

Recently, L-citrulline has been revealed as an efficacious nutritional supplement, capable of affording thermotolerance during HS (Table 1). In chicks and broilers, L-citrulline supplementation was reported to elicit hypothermic effects and thermotolerance [23, 24, 93, 134, 135]. Chowdhury [134] reports L-citrulline as an HS biomarker AA, since its administration was found to induce hypothermia and more so, the systemic concentration of L-citrulline was severely lowered under HS exposure in chickens [136]. Dietary supplementation of 1% L-citrulline to broiler chickens subjected to thermoneutral (24 °C) or HS (35 °C, 60% RH for 5 h) significantly decreased the core body temperature, promoted the antioxidant defenses, and preserved hypothalamic heat shock responses [24]. Orally administered L-citrulline (10 mL/kg body weight) reduced body temperature effectively, validating its hypothermic role in chicks [93]. Similar experiments using watermelon rinds, an agricultural biowaste rich in L-citrulline have corroborated these findings [94, 95]. Also, the utilization of L-citrulline producing live bacterial media persistently lowered the body (surface and rectal) temperature of chicks [96].

**Fig. 7:**
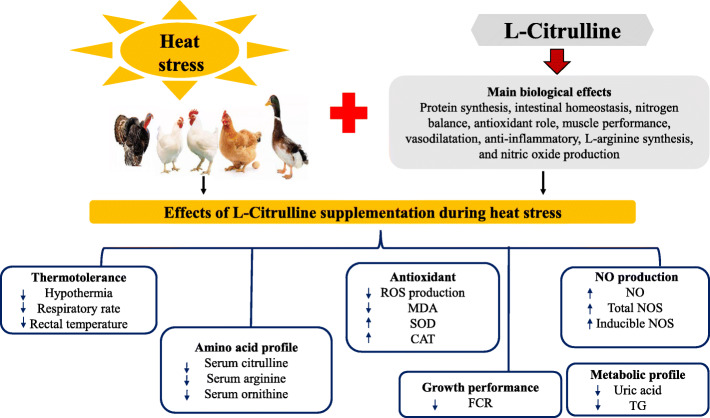
Effects of L-citrulline supplementation during heat stress in animals

In laying hens, dietary L-citrulline supplementation (0.28% L-citrulline) to low protein diets (13% crude protein) did not influence the egg quality, protein digestibility, and energy digestibility of hens, but tended to increase the Haugh unit and lower the egg shell breaking strength [91]. During the summer season, L-citrulline did not affect several parameters related to egg production, egg quality, and plasma biochemistry in laying hens except for an increase in the eggshell index and decreased plasma TG [90]. However, L-citrulline modulated the free serum levels of key AAs involved in arginine metabolism, such as arginine, citrulline, and ornithine [90]. Also, in layer chicks, L-citrulline treatment did not affect the food intake but significantly decreased the plasma glucose levels [23]. Therefore, L-citrulline can be proposed as a novel nutritional candidate that can assist poultry to cope with HS (Fig. 7). In contrast with the findings discussed above, during cyclical HS (33.6 to 38.3 °C), Kvidera [137] reported no significant effects of L-citrulline supplementation (0.13 g/kg BW) on production parameters, although, L-citrulline modestly affected the thermal response by decreasing the respiratory rate, with a tendency to decrease the rectal temperature. In another study, dietary supplementation of 1% L-citrulline did not influence the rectal temperature, feed intake, and body weight but lowered the respiratory rate of sows and pre-weaning mortality of piglets [138]. Similarly, 1% L-citrulline fed to laying hens did not influence the rectal temperature of hens during summer [90]. The reasons for this disparity in findings are yet to be ascertained but point to the differential effects of L-citrulline on body temperature responses which may be dependent on ambient temperature, age and species of birds, and route of administration.

L-citrulline has also been shown to exhibit antioxidative and anti-inflammatory properties. L-citrulline is one of the most potent scavengers of hydroxyl radicals effectively protecting DNA and metabolic enzymes from oxidative injuries [21] and in conditions of nitrosative stress [139]. As a potent antioxidant, L-citrulline acts to scavenge hydroxyl radicals at the rate of 3.9 × 10^9^ M^-1^ s^-1^ [140]. Oral co-administration of L-arginine and L-citrulline lowered superoxide production and oxidation-sensitive genes, *Elk-1* and *p-CREB* expression [141]. Endothelial p67phox expression (an important component of the NADH/NADPH oxidase system) was attenuated by L-citrulline supplementation under high glucose conditions [142]. Also, dietary L-citrulline (2.5 g/kg BW, 6 weeks) provided protective effects to NAFLD mice by lowering markers of liver damage such as hepatic TG, *TLR-4* mRNA expression, neutrophils counts, and plasminogen activator inhibitor-1 protein levels, but promoted the duodenal protein expression of tight junction proteins, occludin and ZO-1 [143]. L-citrulline (200 mg/kg/d) modulated immune functions in infantile rats through lowered neutrophil to lymphocyte ratio, increased IL-10 and TGF-β1 production, and upregulated expression of *SIRT*-1, which plays a regulatory role in the immune system [144].

Furthermore, L-citrulline can stimulate muscle function under stressful conditions [145]. The ability of L-citrulline to maintain muscle mass during protein deficiencies via stimulation of protein synthesis and improvements in muscle functionality has been reported [146–148]. In old malnourished rats, Osowska [149] reported that L-citrulline supplementation increased protein synthesis and muscle protein content, probably by increasing whole-body nitrogen availability, ultimately improving the nutritional status. L-Citrulline supplementation (1 g/kg/d) to food-restricted rats restored the muscle protein synthesis, increased muscle strength, and the maximum tetanic force [150]. In muscle-wasting conditions, Ham [151] demonstrated that L-citrulline could protect protein metabolism and skeletal muscle cell size, independent of L-arginine metabolism. Similarly, Villareal [152] found that L-citrulline supplementation upregulated PGC-1α levels resulting in increased skeletal muscle weights during exercise in rat models. In aged malnourished rats, L-citrulline (5 g/kg/d) supplementation led to higher insulin, liver, and muscle protein mass, higher muscle protein synthesis rate, and improved muscle protein metabolism [149]. More so, it had been reported that L-citrulline regulates muscle protein synthesis via mechanisms of the mTOR pathway [7, 153] and S6K-1 phosphorylation in LPS-treated rats [148]. At present, extensive studies have been conducted on the efficacy of L-citrulline to promote muscle functions and protein metabolism in humans and murine models [7, 130, 131, 146, 153], but this still remains a research gap yet to be explored in poultry species.

#### Betaine

**Fig. 8:**
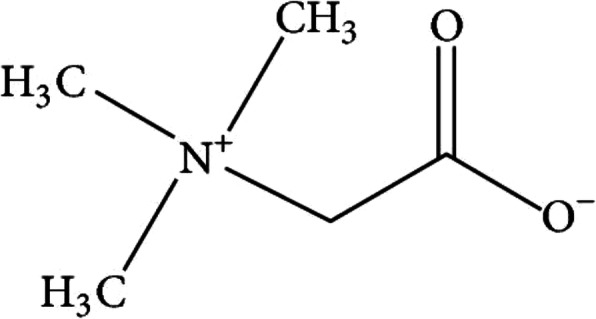
Chemical structure of betaine

**Fig. 9:**
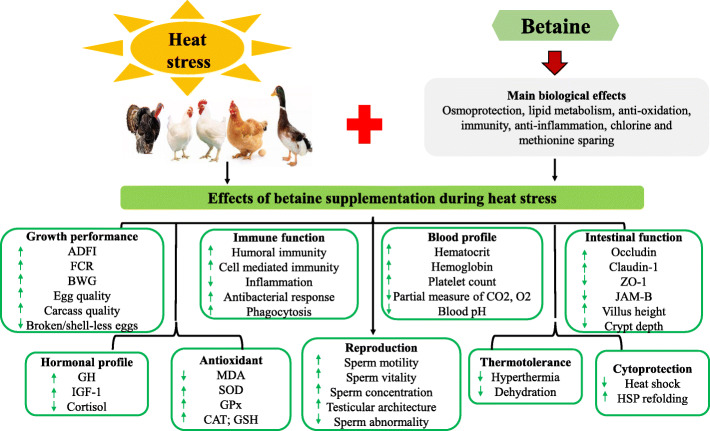
Effects of betaine supplementation during heat stress in animals

Betaine (also known as trimethylglycine betaine) is an amino acid derivative, that is stable, non-toxic, and present in plants, animal tissues, and foods such as wheat, sugar beets, spinach, and aquatic invertebrates [154]. It is a nutritional additive categorized under the functional group of vitamins, pro-vitamins, or chemical substances that have similar effects [27]. Betaine’s structure consists of three methyl groups, from which it can donate methyl groups to the folate pool and also to homocysteine for methionine formation [155] (Fig. 8). Betaine can be synthesized from the oxidation of choline by choline oxidase, and it can also be derived from some feed ingredients as natural sources of betaine [156, 157]. However, the amount and rate of choline synthesis in most animals are inadequate to meet biological demands [158]. In addition, the bioavailability of betaine in feed ingredients such as grains and wheat bran is low [154]. Betaine inclusion in poultry feeds has several benefits (Fig. 9), such as choline and methionine sparing, carcass fat reduction and cell osmoregulation, improved nutrient digestibility, increased growth performance, and feed conversion efficiency in broilers, meat ducks, quails, and turkeys [155, 159, 160]. It is also involved in several biological processes including osmoprotection, lipid metabolism, antioxidation, anti-inflammation, and immunity [159, 161, 162].

Betaine supplemented in drinking water (50 g/kg) or feed (100 g/kg) of HS broilers (36 ± 1 °C, 75% RH; 4 h/d) did not affect the weight gain, feeding and feed/gain ratio, however, the betaine treated chicks were less hyperthermic [28]. Also, in broilers exposed to cyclic temperature conditions (9 h at 28–29 °C and 14 h at 22–24 °C for 2 d), betaine supplementation did not affect the growth or meat quality indices [163]. In contrast, dietary betaine has been shown to improve the average daily gain, European performance index, and humoral immunity of HS (34 °C for 8 h) broilers [86]. It had been suggested that the benefits of betaine may likely be more pronounced when broilers are exposed to temperatures > 32 °C [163], and during stress conditions that affect cell osmolarity [164]. Betaine improved the immune status of HS birds by boosting both humoral and cell-mediated immunity [85, 165]. Klasing [166] explained that betaine’s involvement in promoting cell-mediated immune responses was due to its role in facilitating nitric oxide release from heterophils and macrophages. In addition, betaine was able to decrease inflammation and increase antibacterial response via regulating the osmotic pressure, and phagocytotic activity of monocytes/macrophages [167]. Exposing poultry to HS increases panting for evaporative cooling, water, and electrolyte K^+^ and Na^+^ excretion, and reduces the amount of ionized calcium and bicarbonate levels in the blood [159]. These changes negatively affect the birds, causing acid-base perturbations (known as respiratory alkalosis) [168]. It was reported that the zwitterionic structure of betaine allows for its osmolytic, cytoprotective, and methyl donating functions [157, 169]. Therefore, betaine can alleviate osmotic damage to tissues by preventing dehydration, vascular permeability, loss of blood plasma, and maintaining normal cell volume during hyperthermia [170]. Betaine supplementation to HS ducks (33 to 43 °C; 70% RH, 4 h, d 21-42 ) produced greater BW, improved blood biomarkers, increased the blood electrolyte concentrations, reduced blood gas profiles, and lowered blood pH [88, 171]. Additionally, in female quails, betaine (2 g/kg) promoted the RBC counts but decreased the cortisol, MCV, and MCH, further validating its role in improving erythrocytic parameters [84] and the immune status of HS birds.

Betaine improved performance parameters including the feed intake, egg weight, FCR, protein, energy efficiency ratios, and certain egg quality traits in quails raised at high temperature [172]. Betaine directly promoted the secretion of GH and IGF-1, ameliorating HS depression of growth performance [173]. It was reported that betaine improved the carcass quality, growth, and feed efficiency in ducks, where the dietary methionine levels were not marginally limiting [174]. Similarly, betaine alleviated the adverse effects of long-term HS (32 ± 1 °C, 65-75% RH for 8 h/d; 10 weeks) by improving the BWG, feed intake, nitrogen retention, and intestinal epithelial morphology [87]. In laying hens, dietary betaine decreased the broken and shell-less egg production during HS but it selectively influenced the expression of jejunal tight junction genes [89]. More so, betaine was shown to increase the villus height, villus height to crypt depth ratio, and the protein expression of tight junction proteins in the small intestine of piglets [161]. These studies demonstrate the efficacy of betaine to improve growth performance, carcass quality, immunity, and gut functions during HS conditions (Table 1).

Betaine plays a crucial role in maintaining the antioxidant defense and redox balance during HS. This effect has been attributed to its ability to attenuate mitochondrial lipid peroxidation [175], its protection of the mitochondrial complex [176], modification of cysteine supply in the transsulfuration pathway for GSH synthesis, as well as its regulation of the methionine−homocysteine cycle (to increase methionine and S-adenosylmethionine which forms a protective membrane around cells) [162]. Daily oral supply of betaine (250 mg/kg) induced SOD and GPx activities in HS broilers [83]. Also, the SOD and GPx activity, *CAT, SOD2,* and *GPx* mRNA expression were modulated by dietary betaine supplementation [25]. During the hot-dry season (THI = 27.85-36.1), oral betaine (250 mg/kg) increased GPx and SOD activity, while betaine alone or in combination with ascorbic acid decreased the tonic immobility in broilers [83]. Moreover, the discovery that betaine can decrease fearfulness in broilers was related to its antioxidant-promoting capacity [83]. Dietary betaine (1000 mg/kg) supplemented to HS broilers (34 °C, 8 h for 21 d ) increased the GSH, SOD, GPx activity, but lowered the MDA content in breast muscle of HS broilers [177]. Importantly, betaine also functions in chaperoning, cytoprotection, protein stability, and the cellular regulation of transcription factors [178, 179]. Betaine stabilizes cellular proteins against heat-induced denaturation, as such attenuating and/or inhibiting the induction of heat shock proteins [178]. Diamant [180] showed that betaine increased the rate of HSPs refolding proteins by 30% to 50%, and activated protein disaggregation by 2.5 fold, stabilizing the end product and chaperone structure.

Betaine has also been used in combination with other bioactive substances. Dietary betaine (1000 mg/kg betaine) improved the laying rate, and feed intake of chronic HS hens (38  ± 1 °C; 55% to 65% RH), and demonstrated synergistic effects in combination with Vitamins C and E for HS alleviation [81]. In quails reared during the dry season (THI = 69.8-91.0), dietary betaine and ascorbic acid (Bet (2 g/kg) + AA (200 mg/kg)) enhanced testicular architecture, gonadotropins secretion, sperm motility, gonadal sperm concentration, antioxidant status, but lowered the total sperm abnormalities [181]. In a similar study, Attia [82] showed that the supply of betaine alone (1000 mg/kg), or with vitamin C (200 mg/kg) or vitamin E (150 mg/kg) to chronic HS roosters (38 ± 1 °C; 55–65% RH for 3 d/week) completely reversed the adverse effects of HS on the sperm concentration, sperm livability, semen pH, and fertility, and improved the seminal total antioxidant capacity. Also, in female Japanese quails reared under hot conditions, betaine (2 g/kg) promoted the estradiol levels but decreased the cortisol, further validating the role of betaine in improving serum sex and stress hormones [84]. Betaine enhanced the testicular antioxidant defense and accelerated germinal epithelium regeneration during HS [182]. These improvements of testicular functions by betaine and ascorbic acid are due to the attenuation of oxidative stress and enhanced responsiveness of the hypothalamic-pituitary-gonadal axis for gonadotropin release, and spermatogenesis [82, 164, 181]. Hence, betaine can be utilized as an important additive for inclusion in breeding programs, especially in hot climates since it can exert both antioxidant and protective effects on the reproductive functions of birds.

## Conclusion

HS in poultry is accompanied by a cascade of detrimental effects impacting the behavioral, neuroendocrine, and physiological responses. This review discusses the protective effects of some non-standard amino acids and betaine, and their mechanisms of action in alleviating the detrimental impacts of HS in poultry. These nutrients have been reported to elicit anti-inflammatory, antioxidant, immunomodulatory, and gut-promoting functions in poultry during stress exposure. Commonly utilized as dietary supplements, the incorporation of these nutrients into poultry diets is now considered as a functional additive necessary to improve poultry performance and overall health. It is important to understand that the duration and intensity of HS, poultry nutrition, and variability in HS condition are important considerations when using these nutrients for HS mitigation. Nonetheless, this review provides a better perspective on the less considered AAs that provide functional roles in HS management for poultry and livestock production.

### Future perspectives

Presently, the information available on the actions of taurine, L-theanine, L-citrulline and betaine during HS in poultry are gradually emerging and nonetheless controversial. An understanding of the underlying mechanisms through which they elicit their multiple biological effects is still open for further investigations. Therefore, this creates an existing research gap in poultry nutrition to optimize the utilization and adoption of these nutrients not only under heat stress conditions but also for commercial production practices. More so, it will be interesting to uncover their synergism, imbalance, and antagonistic potentials, when used with the standard AAs for poultry nutrition. Recent advances in biotechnology, molecular biology, and omics technology would together pave ways to strengthen researches in these areas. These tools will be vital in understanding their features, interactions, cross-reactivity, mechanism of action, functional roles, and optimization, ultimately increasing their utilization in health and disease states.

## Data Availability

Not applicable

## Abbreviations

AAs: Amino acids
EAA: Essential AA
NEAA: Non-essential AA
NSAA: Non-standard Amino acids
NAFLD ACC: Acetyl CoA carboxylase
FAS: Fatty acid synthase
HDL-C: High-density lipoprotein cholesterol
HL: Hepatic lipase
HSL: Hormone-sensitive triglyceride lipase
HS: Heat stress
LDL-C: Low-density lipoprotein cholesterol
LPL: Lipoprotein lipase
NEFA: Nonesterified fatty acid
TC: Total cholesterol
TG: Triglycerides
TL: Total lipase
CAT: Catalase
GSH: Glutathione
GPX: Glutathione peroxidase
MDA: Malonaldehyde
SOD: Superoxide dismutase
T-AOC: Total antioxidant capacity
HO-1: Heme oxygenase-1
NRF2: Nuclear factor erythroid 2–related factor 2
AMPKα: Adenosine monophosphate–activated protein kinase alpha
ApoA1: Apolipoprotein A1
SIRT1: Sirtuin 1
CPT-1: Carnitine palmitoyltransferase 1
FAS: Fatty acid synthase
PPARα: Peroxisome proliferator-activated receptor α
SREBP-1: Sterol regulatory element-binding protein-1
MAFbx: Muscle atrophy Fbox protein
MuRF1: Muscle ring-finger protein-1
M-CPT: Muscular isoform of carnitine palmitoyl transferase 1
NAFLD: Non-alcoholic fatty liver disease
ZO-1: Zonula occludens-1, HMOX1 Heme oxygenase 1 gene
Ig: Immunoglobulin
iNOS: inducible nitric oxide synthase
COX-2: Cyclooxygenase
TLRs: Toll-like receptors
DSS: Dextran sulfate sodium
GABAAR: Gamma-aminobutyric acid receptor
